# Improved clinical management but not patient outcome in women with postpartum haemorrhage—An observational study of practical obstetric team training

**DOI:** 10.1371/journal.pone.0203806

**Published:** 2018-09-26

**Authors:** Tinna Baldvinsdóttir, Marie Blomberg, Caroline Lilliecreutz

**Affiliations:** 1 Department of Obstetrics and Gynaecology, and Department of Clinical and Experimental Medicine, Linköping University, Linköping, Sweden; 2 Department of Obstetrics and Gynaecology, Sahlgrenska University Hospital, Gothenburg, Sweden; Indiana University School of Medicine, UNITED STATES

## Abstract

**Objective:**

Postpartum haemorrhage (PPH) is the most common obstetric emergency. A well-established postpartum haemorrhage protocol in the labour ward is crucial for effective treatment. The aim of the study was to investigate if practical obstetric team training improves the patient outcome and clinical management of PPH.

**Setting:**

The practical obstetric team training (PROBE) at Linköping University Hospital, Sweden, with approximate 3000 deliveries annually, was studied between the years of 2004–2011. Each team consisted of one or two midwives, one obstetrician or one junior doctor and one nurse assistant. Emergency obstetrics cases were trained in a simulation setting. PROBE was scheduled during work hours at an interval of 1.5 years.

**Population:**

Pre-PROBE women (N = 419 were defined as all women with vaginal birth between the years of 2004–2007 with an estimated blood loss of ≥1000 ml within the first 24 hours of delivery. Post-PROBE women (N = 483) were defined as all women with vaginal birth between the years of 2008–2011 with an estimated blood loss of ≥1000 ml within the first 24 hours of delivery. The two groups were compared regarding blood loss parameters and management variables using retrospective data from medical records.

**Results:**

No difference was observed in estimated blood loss, haemoglobin level, blood transfusions or the incidence of postpartum haemorrhage between the two groups. Post-PROBE women had more often secured venous access (*p*<0.001), monitoring of vital signs (*p*<0.001) and received fluid resuscitation (*p*<0.001) compared to pre-PROBE women. The use of uterine massage was also more common among the post-PROBE women compared with the pre-PROBE women (*p<*0.001).

**Conclusion:**

PROBE improved clinical management but not patient outcome in women with postpartum haemorrhage in the labour ward. These new findings may have clinical implications since they confirm that training was effective concerning the management of postpartum haemorrhage. However, there is still no clear evidence that simulation training improve patient outcome in women with PPH.

## Introduction

Postpartum haemorrhage (PPH) is the most common emergency in obstetric medicine and one of the leading causes of maternal death. In Sweden PPH is defined as an estimated blood loss of ≥1000 millilitres (ml) after birth regardless of mode of delivery, or as an excessive bleeding causing symptoms or signs of hypovolemia [1]. Some of the causes of PPH are: retained placenta, placental residue in the uterus cavity, and bleeding from vaginal, perineal and/or cervical tears following birth or coagulopathy [2]. However the most common cause for PPH is uterine atony, causing about 80% of the cases [2, 3]. The reported incidence of PPH varies between 1 to 20% and seems to be increasing [3, 4]. The increase in high resource countries may be due to a rising incidence of caesarean section (CS) with a subsequent rise in abnormal placentation [5]. Other possible explanations may be increasing maternal obesity, more widespread use of induction of labour, increasing maternal age when giving birth, and a higher incidence of multiple pregnancies [4, 6]. Since there are known and effective ways to manage and treat extensive blood loss after delivery, PPH is also the most preventable cause of maternal morbidity and mortality [7–9]. Prevention of PPH includes active management in the third stage of labour but also involves recognising risk factors so that uterotonic drugs can be given in advance to certain risk groups [10]. Treatment can vary depending on the cause of PPH, including uterotonic medications, uterine massage and surgical interventions. In clinical practice the blood loss estimation after the delivery is often visual; therefore, the diagnosis of PPH can be uncertain. Visual estimation of blood loss is associated with a 30–50% miscalculation and the underestimation increases with increasing blood loss [11, 12]. It is therefore difficult to recognise PPH with delayed treatment as a consequence. The uncertainty about correctly diagnosing PPH could explain some of the differences in reported incidence.

Recognising an obstetric emergency is the first step in managing the situation. The motivation behind inter-professional team training is to minimize human error and thus improve patient morbidity and mortality [13]. Team training is aimed at early recognition of an emergency, early initiation of correct treatment, and enhancing team communication and emergency skills in situations that do not often occur in clinical practice. Implementation and development of structured simulation-based training in obstetric medicine was presented in 1990 and in 1991 Advanced Life Support in Obstetric (ALSO®) was introduced in the United States [14]. Since then, a variety of different training programs have been developed and many training programs have been designed to allow training at individual maternity units [15]. After the introduction of different training programs, the effect has been evaluated in multiple studies. Simulation-based team training has been shown to increase group efficiency and communication and emergency skills, and to strengthen the staffs’ self-efficacy [13, 15, 16]. To evaluate whether patient outcome has improved with the initiation of training programs has proven to be more of a challenge and the results of such studies are diverse [17, 18, 19].

The aim of this study was to evaluate whether the patient outcome and clinical management among women with PPH had improved after practical obstetric team training (PROBE) compared with patient outcome and clinical management before PROBE.

## Materials and methods

The study was approved by the Regional Ethics Committee for Human Research of the Faculty of Health Sciences, Linköping University Dnr 2016/177-31. The ethics committee decided that informed consent was not needed from the study population as all data were coded and presented anonymously.

The obstetric department Linköping University Hospital (US), Sweden, has approximately 3000 deliveries annually. All women giving birth at US in gestational week ≥23+0 and who had an estimated bleeding of ≥1000mL within the first 24 hours of delivery were collected. To identify women with PPH during the period 2004–2011 all medical records with the ICD-10 diagnoses of O70, O71, O72 and O75 were extracted from the hospital’s electronic birth- and maternity care registration system Obstetrix ®. The content and the registered variables in the electronic medical record have been identical during the whole study period. The documentation in Obstetrix ® was performed by the responsible midwife and the obstetrician on duty. Each woman’s medical records were then individually scrutinized by the first author to confirm the diagnosis and the estimated blood loss of ≥1000mL and to extract relevant data into a standard data extraction form. In those cases where blood transfusion was not documented in the women’s records the transfusion notes were also examined. If the ICD- 10 diagnose was not confirmed by the data in the medical records the women was excluded from the study.

The following variables were extracted: age, parity (0, 1, >1), gestational age, body mass index (BMI <20, 20–24, 25–29, 30–34, 35–39, >40), gestational hypertension or pre-eclampsia (yes/no) and history of previous PPH (yes/no), if the labour was induced (yes/no), if the contraction were stimulated with oxytocin (yes/no), the length of active labour, defined as when the patient was considered to be in active labour until delivery, was divided into less than 10 h and more than 10h, Delivery method (partus normalis (PN), vacuum extraction (VE) or (CS)) were extracted. Estimated blood loss, which during the study period was visually estimated (pre-PROBE) or weighed (post-PROBE) by the midwife or doctor, was documented along with the lowest postpartum haemoglobin (Hb) level measured in g/L. Red blood cell (RBC) transfusion or not before discharge from the clinic was noted. Clinical management variables collected from the women’s records were: venous access defined as at least two working venous catheters at the time of hemorrhage (yes/no), infusion of crystalloids or colloids (yes/no), uterine massage (yes/no) and monitoring of the patient’s vital signs (yes/no). Data was collected on given uterotonic medications as well as in what order the medications were given when a patient received more than one drug. Medications used to manage PPH were oxytocin bolus injection of 8.3 micrograms (μg) (1IU) (yes/no), Misoprostol usually given as a dose of 0.8 milligram (mg) per rectum (yes/no), methylergometrine 0.2 mg administered intravenously (yes/no), carboprost 0.25 mg given intramuscularly (i.m) (yes/no) and oxytocin infusion (24.9 μg (30 IU) oxytocin in 500 mL 5% glucose solution) (yes/no). Surgical interventions extracted were: uterine balloons (yes/no), manual retrieval of the placenta (yes/no), curettage (yes/no), B-Lynch sutures (yes/no) and hysterectomy (yes/no). All extracted variables concerning the event of PPH were documented in the medical records including the timing of the interventions related to PPH; it was therefore possible to identify the temporal sequence of events.

In 2008, a simulation-based team training program, practical obstetric team training (PROBE), was introduced at the delivery ward in Linköping University Hospital Sweden (US). Responsible for the content of PROBE curriculum is the head of the department of obstetrics (co-author MB) in collaboration with the instructors. The objective was to improve inter-professional teamwork and obstetric emergency skills and thus improve patient outcome and clinical management. PROBE is mandatory for doctors in the field of obstetrics, midwives and nurse assistants that work at the delivery ward and is scheduled during work hours at an interval of 1.5 years. PROBE training sessions are held at a simulation centre of US, where there is an obstetric skills laboratory. Each training session is three hours long and includes two simulation scenarios and one practical skills training. PROBE is held by trained instructors, both midwives and doctors, all of whom have gone through ALSO® training, and the PROBE training is based on the concept of ALSO®. During PROBE, obstetric emergencies are simulated using either actors, usually instructors, or training mannequins depending on the scenario. In each simulation, staff members work in teams of one or two midwives, a doctor and a nurse assistant so that the simulation is as realistic as possible. Instructors observe the team during each simulation. Focus during training is recognition of an emergency and management of the emergency using as standardized methods as possible, similar to the algorithms used during cardio-respiratory resuscitation (CPR). These management methods are the same as those in the obstetric department’s clinical guidelines. PROBE also focuses on teamwork and communication. After a simulation scenario the team along with the instructors perform a systematic evaluation of the exercise. The evaluation consists of three parts; first everyone reconfirms what happened, second everyone says what they did well, and finally everyone summarizes what they learned and will take with them to clinical practice. PROBE included the management of PPH as a simulation scenario during the study period.

The women were divided into two study groups, those with PPH during 2004–2007 before PROBE was introduced, defined as pre-PROBE women, and those women with PPH during 2008–2011,defined as post-PROBE women.

Analyses of background data was performed. Women with PPH and CS where then excluded from further analyses and the final study population consisted of women with PPH and vaginal delivery.

The primary aim was to investigate if PPH patient outcomes had changed regarding estimated blood loss, postpartum Hb levels and the presence of RBC transfusions before discharge. A secondary aim was to investigate if the clinical management of PPH had changed regarding venous access, uterine massage, fluid resuscitation, monitoring of vital signs, administration of uterotonic medications, manual removal of the placenta, as well as other surgical interventions to stop PPH.

### Statistical analyses

All analyses were performed using the SPSS program 22.0 (IBM Inc., Armonk, NY). Statistical significance was defined as two-sided P-values using a significance level of 5%. The t-test was used for quantitative variables with approximately normal distribution and Chi-Square tests were used when analysing the categorical variables. BMI, oxytocin stimulation, delivery method (partus normalis, vacuum extraction) and length of active labour >10h were considered as putative confounding factors and were thus included in the adjusted analysis through multiple logistic regression analyses comparing pre-PROBE to post-PROBE outcomes.

## Results

During the years of 2004–2007, the cumulative incidence for PPH was 4.5% (450/9906), and the 95% CI was 4.1%-5.0%. The corresponding incidence for PPH for the time period 2008–2011 was 4.7% (493/10.487) where the 95% CI was 4.3%-5.1%. There was no difference in the incidence of PPH between the time periods (p = 0.496).

After PROBE was introduced women with PPH (n = 493) more often had a normal vaginal delivery and were less likely to be delivered by CS (2.0% vs 6.9%, (p<0.001)) compared to women with PPH before PROBE (n = 450). The same result were found in all women giving birth; a normal vaginal delivery increased between the period 2004–2007 comparing to the period 2008–2011 (p = <0.001). Since there was a difference in the rate of CS in PPH women before and after PROBE and the management of PPH may differ between CS compared to a vaginal delivery the final study population only included women with a vaginal delivery. The distribution of background and obstetric data of the women included in the study is shown in Table 1. The same analysis of background data was performed including also patients with PPH and CS. The results were the same as the one shown in Table 1 (data not shown).

**Table 1:** Background and obstetric characteristics. Including only women with a vaginal delivery.

		2004–2007 (n = 419)		2008–2011 (n = 483)		
		n	%	N	%	p-value
**Maternal age (years), mean/SD**		30.00/4.6		30.07/5.147		0.81
**BMI (kg/m**^**2**^**)(n = 393/470)**	>20	11	3.0	29	6.3	0.004
	20–24	192	52.0	230	49.8	
	25–29	105	28.5	145	31.4	
	30–34	41	11.1	42	9.1	
	35–39	18	4.9	7	1.5	
	>40	2	0.5	9	1.9	
**Nulliparous**	Yes	207	49.4	241	49.9	0.95
**Gestational age (weeks), mean/SD**		39.38/1.92		39.30/2.26		0.55
**Previous PPH**	Yes	47	11.3	49	10.2	0.59
**Hypertension/Pre-eclampsia**	Yes	21	5.0	27	6.7	0.30
**Singleton**	Yes	412	98.3	469	97.1	0.37
**Induction of labour**	Yes	78	18.6	102	21.1	0.35
	No	3411	81.4	381	78.9	
**Oxytocin during labour**	Yes	236	56.3	230	47.6	0.009
	No	183	43.7	253	52.4	
**Length of active labour**						
**0–10 hours**	Yes	379	90.5	402	84.1	0.005
**>10 hours**	Yes	40	9.5	76	15.9	
**Epidural analgesia**	Yes	141	33.7	156	38.9	0.10
**Delivery method**	PN	344	82.1	408	84.5	0.34
	VE	75	17.9	75	15.5	

Standard deviation (SD) is given with mean, and percentage (%) is given with the number of patients. BMI, body mass index. PPH, postpartum haemorrhage. PN, partus normalis. VE, vacuum extraction

The pre-PROBE women had a different distribution of BMI compared to the post-PROBE women (*p* = 0.004). The post-PROBE women had a longer length of active labour (>10 hours) compared to the pre-PROBE women (*p =* 0.005). Among the post-PROBE women, the use of oxytocin to stimulate contractions during labour was less common compared to the pre-PROBE women (*p* = 0.009).

Table 2 shows the PPH variables among vaginally-delivered women.

**Table 2:** PPH and management variables, including only vaginal deliveries.

		2004–2007 (n = 419)		2008–2011 (n = 483)		
		n	%	n	%	p-value
**Estimated blood loss (mL), mean/SD**		1632.5/572.2		1738.6/868.9		0.03
**Hb (g/L)**	<100	286	68.3	337	69.8	0.62
	<80	109	26.0	120	24.8	0.69
**RBC transfusion**	Yes	125	29.8	158	32.7	0.35
	No	294	70.2	325	67.3	
**Venous access**	Yes	393	93.8	481	99.6	<0.00
	No	26	6.2	2	0.4	
**Infusion of colloids/crystalloids**	Yes	363	86.6	469	97.1	<0.00
	No	56	13.4	9	1.9	
**Uterine massage**	Yes	378	90.2	471	97.5	<0.00
	No	38	9.1	8	1.7	
**Monitoring of vital signs**	Yes	359	85.7	470	97.3	<0.00
	No	58	13.8	7	1.4	
**Administration of uterotonics**	None	7	1.6	4	0.8	<0.00
	1	141	33.7	81	16.8	
	2	167	39.9	160	33.1	
	3	86	20.5	142	29.4	
	4	18	4.3	96	19.9	
**Manual removal of placenta**	Yes	164	39.1	209	43.5	0.32
	No	173	41.3	180	37.4	
	Other surgical intervention	82	19.6	92	19.1	

Standard deviation (SD) is given with mean and percentage (%) is given with the number of patients. RBC transfusion, red blood cell transfusion.

The estimated blood loss was higher in the post-PROBE women compared to the pre-PROBE women (*p* = 0.033). A multiple logistic regression did not confirm that result. Hb levels and the administration of RBC transfusions after delivery were the same in both study groups (*p* = 0.624, 0.353). Post-PROBE women had more often secured venous access (99.6% (CI 99.0%-1.00%) vs 93.8% (CI 91.6%-96.0%)) (*p*<0.00), monitoring of vital signs (97.3% (CI 95.9%-98.7%) vs 85,7% (CI 84.4%-90.5%)) (*p*<0.00) and more often received fluid resuscitation with infusions of colloids/crystalloids (97.1% (CI 95.6%-98.6%) vs 86.6% (CI 83.5%-89.7%)(*p*<0.00)) compared to pre-PROBE women. The use of uterine massage was also more common among the post-PROBE women compared with the pre-PROBE women (97.5% (CI 96.1%-98.9%) vs 90,2% (CI 87.5%-92.9%)) (*p <*0.001).

The number of uterotonic medications given was higher in the post-PROBE women compared to the pre-PROBE women (*p*<0.001) and the use of Oxytocin (bolus injection and infusion) and Misoprostol increased in the post-PROBE women group (*p*<0.001).

In Table 3 the most commonly used uterotonic medications as first, second, third and fourth choice, are presented in given order.

**Table 3:** Most common uterotonic medications in the order they were given, including only vaginal deliveries.

	2004–2007 (n = 419)			2008–2011 (n = 483)			
		n	%		n	%	p-value
**First given uterotonic**	Oxytocin bolus injection	183	43.7	Oxytocin bolus injection	281	58.2	<0.00
**Second given uterotonic**	Oxytocin infusion	130	31.0	Misoprostol	136	28.2	<0.00
**Third given uterotonic**	Misoprostol	44	10.5	Misoprostol	77	15.9	<0.00
**Fourth given uterotonic**	Misoprostol	8	1.9	Oxytocin infusion	33	6.8	<0.00

In Table 4, results are shown from the multivariable logistic regression analyses using the post-PROBE women group as a dependent variable, and adjusting for possible confounders (BMI, oxytocin stimulation, delivery method and length of active labour >10h).

**Table 4:** Multivariable logistic regression analyses comparing pre-PROBE to post-PROBE outcome. Including only vaginal deliveries.

	aOR (95% CI)	p-value
**RBC transfusion**	0.87 (0.64–1.18)	0.32
**Hb <100**	0.93 (0.68–1.26)	0.64
**Hb<80**	1.08 (0.78–1.49)	0.65
**Blood loss >2000mL**	0.87 (0.63–1.20)	0.41
**Blood loss >1500mL**	0.95 (0.72–1.26)	0.72
**Surgical intervention**	0.86 (0.64–1.15)	0.33
**Uterine massage**	5.78 (2.65–12.60)	<0.00
**Infusion (colloid/crystalloid)**	8.52 (4.13–17.60)	<0.00

Adjustments were made for possible confounders (BMI, oxytocin stimulation, delivery method (partus normalis or, vacuum extraction) and length of active labour >10h). OR, odds ratio, CI, confidence interval. RBC transfusion, red blood cell transfusion.

No differences between the study groups concerning estimated blood loss, Hb levels or RBC transfusions were found. The post-PROBE women were more likely to receive uterine massage (*p <*0.001) and infusion of colloid/crystalloids (*p* <0.001) compared to the pre-PROBE women.

## Discussion

This study did not find any improvement concerning PPH patient outcome in relation to estimated blood loss, Hb levels after PPH and RBC transfusions given after the introduction of PROBE. After PROBE training women with PPH were more likely to receive secure venous access, fluid resuscitation, vital sign monitoring and uterine massage. A significant change in the use of numbers and types of uterotonics after PROBE was also observed. The incidence of PPH remained unchanged. If patient outcome in future studies cannot be confirmed, there is a risk that different training programs will start to be questioned.

One might speculate whether PROBE training prevents some cases of PPH because even though the increased length of labour (length of active labour >10h) and maternal obesity, both with increased risk of uterine atony were more common in the post-PROBE women the incidence of PPH and estimated blood loss remained the same. The change in PPH clinical management after PROBE suggests that the delivery ward staff were better trained in clinical management of PPH and that they worked in a more standardized way when encountering PPH. Ensuring that the women has venous access, receives fluid resuscitation, monitoring of vital signs, and uterine compression, and that uterotonics are administered in a correct manner could increase maternal safety.

Other studies using Hb levels and RBC transfusions as substitute markers for blood loss have shown mixed results. A Norwegian study compared women with a blood loss >500mL one year before a mandatory training program was implemented with a group of women with a blood loss >500mL one year after. Their study did not find a significant change in incidence of PPH, estimated blood loss or Hb levels. However, they observed a significant decrease in RBC transfusions, with 20.8% of women receiving transfusions during the year before training compared to 12.3% after training (*p* <0.001) [18]. Differences in the definition of PPH (>500mL vs >1000mL) and possible differences in transfusion routines could explain the different results. Another study including 148 deliveries, 50 of them CS, did not observe a change in RBC transfusions or Hb levels in women with PPH after vaginal delivery; however, a decrease in RBC transfusions after CS with PPH was noted after the implementation of a training program [19]. This study had a smaller study population as well as a higher incidence of CS than our study. To our knowledge there is yet no published study on patient clinical management of PPH.

In our study, post-PROBE women were given more uterotonics, which indicates a more aggressive treatment with uterotonic medications after PROBE was introduced. The PROBE concept and the postpartum haemorrhage protocol in the clinic were in agreement with that finding. According to the local protocol used in the delivery ward, midwives are able to administer some uterotonics before the on-call physician arrives. This could possibly prevent some cases of PPH and limit blood loss. Oxytocin was, during the whole study period, the most common first uterotonic to be given, and was given as an extra bolus injection in addition to the bolus all women should receive according to the clinic’s guidelines. Oxytocin is considered the first line uterotonic and is probably the most potent one [8]. After PROBE was introduced, the uterotonic misoprostol was more frequently used. This result reflects that the PROBE training increased compliance to the clinic´s postpartum haemorrhage protocol. Although there was evidence of an association between team training and improved clinical management of PPH cases, the amount of estimated blood lost Hb levels after PPH and RBC transfusions did not differ following training. It is in a way problematic that worldwide team training of emergency obstetric event takes place, including PPH training, without clear evidence of benefits for the mother. Especially in the light of limited financial resources and staff participants’ time taken from basic clinical care. Results presented in this study must though be interpreted with caution since the number of vaginal deliveries in the total pregnant population significantly increased between the pre- and postPROBE periods. We can only speculate upon whether there was an increase in the number of lengthy labors among the total pregnant population postPROBE compared to prePROBE There is therefore a possibility that findings in this study of an unchanged rate of PPH is actually an improvement.

An advantage of this study is the fact that we reached the total pregnant population at the time of the study and that data were extracted from the standardized medical records and not from maternal recall. It is possible that some women with PPH did not receive an ICD code of PPH in the medical record properly, and thus were not found when making the first selection based on ICD codes. That problem exists for both groups (pre-PROBE, post-PROBE) but may lead to an underestimation of the incidence of PPH. Another potential weakness is that the medical records, from which all information was drawn, despite standardization, sometimes lack some information. However, this dilemma exists in both groups and is therefore unlikely to have affected the results. Documentation routines were not changed during the PROBE team training, but it is possible that the training itself have led to an increase in documentation which might have altered the results. Another main strength is the fact that PROBE training was mandatory, ensuring that all personnel had been through training with simulation of PPH with an interval of 1.5 years. We also consider it a strength that the study spans an extended period, allowing changes in routines to be implemented in clinical praxis.

One limitation of this study is that the estimation of blood loss after the delivery was done visually by a midwife or a doctor in the pre-PROBE group and by weighing the equipment used during delivery in the post-PROBE group. Therefore, the diagnosis of PPH and the amount of blood loss in the pre-PROBE group may be more uncertain as visual estimation of blood loss is usually underestimated [20]. However, it has been shown that training in estimation of blood loss increases estimation accuracy [21]. Estimated blood loss might therefore have been more accurate after PROBE was introduced. There is no difference in the estimated blood loss over the whole study period and one might speculate whether the estimation of blood loss before PROBE was less accurate, preventing us from showing an improvement in estimated blood loss due to PROBE. The measurements of Hb and units of RBC transfusion given after birth were therefore substitute markers for actual blood loss, but no differences according to those variables were found. The clinic did not change the guidelines for when women should receive RBC transfusions after birth during the study period.

Another limitation is that it is impossible to determine if other changes in the routines of the delivery department had an impact on the results of the study.

The present study population consisted of women with PPH, therefore other possible effects, of the PROBE training on all women giving birth, for example averted postpartum haemorrhages, could not be evaluated.

## Conclusions

In this study we could not confirm improvement regarding patient outcome i.e clear evidence of benefits for the mother. The new finding that post-PROBE women were more effectively clinical managed was confirmed by the results that these women were monitored to a greater extent than pre-PROBE women. In the light of limited financial resources and staff participants’ time taken from basic clinical care the effects of team training needs to be further addressed.
